# Assessment of Insulin Injection Practice of Nurses Working in a Tertiary Healthcare Center of Nepal

**DOI:** 10.1155/2018/9375067

**Published:** 2018-08-01

**Authors:** Shital Adhikari, Ramesh Sharma Poudel, Laxmi Rajbanshi, Shakti Shrestha

**Affiliations:** ^1^Department of Medicine, Chitwan Medical College Teaching Hospital, Chitwan, Nepal; ^2^Department of Nursing, Chitwan Medical College Teaching Hospital, Chitwan, Nepal; ^3^Department of Pharmacy, Chitwan Medical College Teaching Hospital, Chitwan, Nepal

## Abstract

**Introduction:**

Sound knowledge and good practice on insulin injection technique are essential for nurses in order to administer insulin correctly and to educate patients or their relatives adequately. This study aimed to assess the insulin injection practice through the use of insulin pen among nurses working in a tertiary healthcare center of Nepal.

**Materials and Methods:**

A cross-sectional descriptive study was conducted among 67 nurses working in one of the tertiary healthcare centers of Nepal. Demographic information and insulin injection practice of nurses through the use of insulin pen were assessed using self-administered questionnaire. Each correct practice was scored “1” and incorrect practice was scored “0.”* Results.* The median (IQR) insulin injection practice score of nurses was 11 (9-12) out of 16. Thirty-seven (55.2%) nurses store insulin pen filled with insulin cartridge at room temperature while 57 (85.1%) nurses store unopened cartridge at refrigerator (2-8°C). The practice of hand washing and injection site cleaning was mentioned by 92.5% and 82.1% of the nurses, respectively. However, just over half of the nurses mix the premix (cloudy) insulin and prime insulin pen before each injection. Thirty-four (50.7%) nurses do not lift skin during injection and more than half of the nurses keep needle beneath the skin for less than 5 seconds after completely injecting the required dose of insulin. One out of ten nurses massage injection site after injecting insulin. Most of the nurses (86.6%) use single needle more than once and the median (IQR) frequency of needle reuse was 6 (3-12). Similarly, systematic site rotation was performed by 59 (88.1%) nurses and twenty (29.9%) nurses claim that they use single insulin pen for two different cartridges.

**Conclusion:**

The insulin injection practice of nurses assessed through the use of insulin pen was suboptimal and highlights the need for urgent educational intervention.

## 1. Introduction

Diabetes mellitus (DM) affects 425 million people worldwide and about three-quarters of them live in low- and middle-income countries [1]. Prevalence of diabetes in Nepal is between 4.1% and 9.5% [2], with urban areas reporting higher prevalence rates [3]. Appropriate glycemic control is necessary for improving clinical outcomes and reducing mortality in hospitalized patients [4]. Therefore, correct administration of insulin and oral medications is crucial to managing hyperglycemia and inappropriate technique may result in adverse health outcomes [4–8]. As frontline healthcare professionals, nurses are responsible for injecting insulin and educating patients about correct insulin injection technique in inpatient setting. Therefore, they should have sound knowledge and good practice on insulin injection technique to administer insulin correctly and to educate patients or their relatives properly. However, study in Northern Ireland demonstrated that district nurses' knowledge and practice relating to insulin therapy was insufficient [9]. Similarly, study in Jordan showed that registered nurses had insufficient knowledge on insulin storage and preparation [10]. Likewise, studies from Pakistan [11] and Greece [12] also demonstrated that nurses have inadequate knowledge on insulin injection technique. Such inadequacies may cause insulin administration errors and adverse outcomes. In Nepal, continuing professional development (CPD) program for nurses are limited and it is not mandatory for them to participate in such program. Also, post-registration training for renewal of license is not required for them [13]. Moreover, training related to evidence-based practices is rarely provided to Nepalese nurses [14]. These might be the contributing factors responsible for incompetencies in some areas of nursing services, if not all [15–18]. Additionally, commercialization of the profession is strongly attributed to deteriorating nursing education and practice in Nepal [19]. In recent years, insulin pen is preferred over traditional syringes and vials for insulin administration [20, 21]. Insulin pen is strongly recommended by the healthcare professionals and also preferred by the patients [22]. Studies have demonstrated that nurses also prefer the use of insulin pen to vial and syringe for various reasons (e.g., easier to measure insulin dose, easier to teach patients, less time required for preparation and administration, etc.) [23–25]. However, taking into account the aforementioned circumstances, Nepalese nurses may not be competent to use insulin pen in clinical settings.

Assessment of nurses' insulin injection practice is important to ensure the safe and effective use of insulin pens. Therefore, it is necessary to assess the insulin injection practices of nurses from Nepal where an evidence of such practice is lacking. The appropriately collected data will provide useful information for developing interventional strategies in order to improve current practice. The objective of this study is to assess the insulin injection practice through the use of insulin pen among nurses working in a tertiary healthcare center of Nepal.

## 2. Materials and Methods

This was a cross-sectional descriptive study conducted among nurses working in one of the tertiary healthcare centers of Nepal in April 2018. Ethical approval of this study was obtained from the Institutional Review Committee of the participating hospital. Two hundred eighty-five nurses were working in different departments/units of hospital during this study period. Among them, eighty-five nurses working in Operation Theater, Pediatric ward, Neonatal Intensive Care Unit, Pediatric Intensive Care Unit, and Emergency Department were excluded because these nurses do not usually administer insulin subcutaneously using insulin pen. Out of 200 nurses, 68 of them who were working on morning shift during the day of data collection were selected purposefully to reduce bias. One of the nurses refused to participate, therefore providing only 67 valid sets of data. Self-administered questionnaire was developed to assess insulin injection practice of nurses through insulin pen and was based on new insulin delivery recommendation [26]. The recommendations were transformed into questions taking into account the current circumstances in which the questions were to be applied such as unavailability of 4 mm needle which does not require skin lifting and mandatory site cleaning because we were assessing the insulin injection practice of nurses in hospital setting which would otherwise be optional in noninstitutionalised setting and cover basic components of insulin injection technique through insulin pen. Then questionnaire was pilot tested among ten nurses working in Emergency Department of the same hospital. These nurses were requested to complete the questionnaire individually and were asked for feedback on the clarity of the questions. Accordingly, the questionnaire was refined. Section one consisted of demographic characteristics: age (in years), qualification, working areas, overall working experience (in years), and previous class/training on insulin injection technique through insulin pen (Yes/No). Section two included 17 questions focusing on insulin injection practice using insulin pen: storage of insulin pen filled with cartridge, storage of unopened cartridge, different time gaps based on meals for different types of insulin, hand washing prior to injection, cleaning of injection site, mixing of premix insulin prior to each injection, priming of insulin pen prior to each injection, insulin injection sites, lifting of skin during injection, angle of insulin injection, holding time, massage of insulin injection site, length of needle, reuse of needle, needle reuse frequency, systematic site rotation, and use of single insulin pen for two different cartridges. A trained research assistant met the nurses in their working stations, described the purpose of study and requested to complete the questionnaire after taking their verbal consent. The participants were requested to fill up the questionnaire in the presence of the research assistant in order to avoid external influences and to clarify the questions. Participant confidentiality was maintained throughout this study. Each correct practice was scored “1” and incorrect practice was scored “0.” The highest possible score in this study was 16 as we did not include the score for needle reuse frequency. Therefore, the nurses who scored “16” were considered optimal for insulin injection practice using insulin pen. Descriptive statistics were performed using IBM-SPSS 20.0 (IBM Corporation, Armonk, NY, USA). Numeric variables (age, working experience, needle reuse frequency, and insulin injection practice score) were tested for normality using Shapiro-Wilk test. However, as all failed to follow normal distribution, median and interquartile range (IQR) was calculated. The categorical variables were presented as frequency and their respective percentage. Kruskal-Wallis test was performed to observe difference in insulin injection practice score with respect to qualification of nurses.

## 3. Results

Of 68 nurses, 67 (98.52%) nurses gave consent to participate in this study. The median (IQR) age of the participants was 23 (21-24) years (ranged from 18 to 35 years). Majority of the nurses had completed Proficiency Certificate Level in Nursing (61.2%) while 19 (28.4%) nurses had completed Bachelor of Science in Nursing (28.4%) and seven (10.4%) nurses had completed either Bachelor of Nursing or Bachelor of Nursing Sciences. Thirty-four nurses (50.7%) were working in general wards and the remaining nurses were working in critical care units. The overall median (IQR) working experience of the participants was 1 (0.75-2) year (ranged from 0.25 to 6 years). About four-fifth of the nurses claimed that they had not attained any formal class or training to use insulin pen.

The median (IQR) insulin injection practice score was 11 (9-12) out of 16. Assessment of insulin injection practice of nurses using insulin pen revealed that 37 (55.2%) nurses store insulin pen filled with insulin cartridge at room temperature while 57 (85.1%) nurses store unopened cartridge at refrigerator (2-8°C). Fifty-four (80.6%) nurses told that they were familiar with different types of insulin and their administration schedule with respect to meal. Hand washing and injection site cleaning were practiced by 62 (92.5%) and 55 (82.1%) nurses, respectively. However, mixing of premix (cloudy) insulin and priming of insulin pen prior to each injection was performed by 35 (52.2%) and 34 (50.7%) nurses, respectively. Abdomen and thigh were the most common sites chosen for injection and arms were also used by five (7.5%) nurses. Thirty-four nurses (50.7%) do not lift skin during injection. Forty-four (65.7%) nurses inject insulin nearly at 90° and thirty-five (52.2%) nurses keep needle beneath the skin for less than 5 seconds after injecting required dose of insulin. One out of ten nurses reported that they massage injection site after injecting insulin. Only eight nurses mentioned the appropriate length of needle for insulin pen. Fifty-eight nurses (86.6%) used one needle more than once and median (IQR) frequency of needle reuse was 6 (3-12). Similarly, 59 (88.1%) nurses claimed that they do systematic site rotation while injecting insulin. Twenty (29.9%) nurses mentioned that they use single insulin pen for two different cartridges. The median (IQR) practice score for nurses who completed Proficiency Certificate Level in Nursing, Bachelor of Science in Nursing, and Bachelor of Nursing or Bachelor of Nursing Sciences was 10 (9-12), 11 (9-12), and 11 (9-14), respectively. However, this difference was not statistically significant (p = 0.347).

## 4. Discussion

As key frontline healthcare professionals, nurses are responsible for proper insulin administration for hospitalized patients with diabetes. Therefore, their knowledge, practice, and commitment are key factors for controlling patients' blood sugar levels and for educating patients and their relatives for correct insulin injection technique through insulin pen. However, present study showed inconsistency between insulin injection practice of nurses using insulin pen and new insulin delivery recommendation [26]. This is probably due to lack of formal classes or training on insulin injection technique through insulin pen as reported in this study. Similarly, the present study demonstrated that there was no statistically significant difference in insulin injection practice score with respect to qualification of nurses. In the context of Nepal, specialty training on newer medical devices such as insulin pen is rare and mostly depends upon individual rather than on mandatory training provision in nursing education itself or through CPD program. Moreover, in this study, involvement of younger and less experienced nurses might be responsible for such suboptimal practice. Retention of nurses in private hospital and nursing homes is challenging in Nepal. Turnover of junior nursing staff remains high in major urban hospitals [27]. Moreover, report suggests that it is challenging to retain nurses even in government hospitals of Nepal. Reasons described include better job opportunities in other hospitals of Nepal, going abroad for further study and sometimes even due to personal reasons (e.g., being married in different location) [27, 28]. In addition, majority of the Nepalese nurses have been known to work under stress because of inadequate staff supply and lack of properly functioning equipment which further contribute to high turnover rates of nurses in Nepalese hospitals [29]. However, there is no effective management strategy for retaining senior nursing workforce in Nepalese hospitals [27]. This results in younger and less experienced nurses in the healthcare system of Nepal. Similar cases of insufficient knowledge of practicing nurses on insulin administration were observed in Northern Ireland [9], Pakistan [11], Greece [12], and United States [30]. Other studies in Jordan [10] and United States [31] also reported that nurses had inadequate knowledge on insulin.

It is recommended that unopened insulin cartridge should be stored in a refrigerator (2-8°C) while insulin pen in use can be stored at room temperature (15-30°C) for up to 30 days [26]. However, healthcare professionals should check the manufacturer's recommendation for proper storage condition [32]. But 44.8% of nurses in this study stored insulin pen in refrigerator and 14.9% of nurses stored unopened cartridge at room temperature. Storage of insulin pen with cartridge inside in refrigerator and its subsequent transfer at room temperature (cause temperature variation) leads to accumulation of air in the pen which prolongs the insulin delivery time and increases the risk of underdosing [33]. Similarly, storage of insulin at elevated temperature decreases the potency of insulin [34]. Majority of nurses in this study reported that they inject different insulin preparations at different time intervals as per meals consumed and as recommended. However, studies demonstrated that variation in injection-to-meal time causes no change in HbA1c level [35, 36]. Insulin should be injected into a clean site using clean hands and the use of disinfectant is recommended in institutional settings such as hospitals and nursing homes [26]. However, some of the nurses in the present study did not follow this recommendation while injecting insulin. This practice may increase the risk of contamination and infection. Just below half of the nurses did not resuspend premix (cloudy) insulin prior to each injection in the present study. Such practice causes inadequate resuspension of insulin leading to varying concentration of insulin within the same cartridge and hence unpredictable clinical responses [26]. Moreover, this practice also leads to higher consumption of insulin [37]. Priming of insulin pen is necessary to facilitate the removal of air bubbles, which improves the accuracy of the insulin dose [38]. However, half of the nurses were found not to prime the insulin pen before each injection in this study. The purpose of lifting a skinfold is to increase the distance from the skin surface to the muscle and this practice was followed by half of the nurses in this study. Lifting a skinfold is rarely needed if abundant subcutaneous tissue is present in injection site [26]. In this study, more than half of the nurses mentioned that they would keep the needle under the skin for less than 5 seconds after completely inserting the required dose of insulin. This insufficient holding time causes leakage of insulin from insulin pen [26]. In this study, we found that six nurses were unaware that injection site should not be massaged after injection and this practice is not compatible with current recommendation [26]. Needle size is also important for insulin injection and 4 mm needle is considered the safest size. However, 6 mm needle may be used by lifting skinfold and injecting insulin at 45° angle [26]. Appropriate length of needle was mentioned by few nurses in this study. Previous study in the same setting has demonstrated that insulin needle length of 6 mm is most commonly used by patients while the use of 4 mm needle was not reported, possibly due to its unavailability in Nepalese market [39]. Therefore, our study considered skin lifting as a mandatory step while using insulin pen, which would, however, make no difference in the delivery of insulin using a 4 mm needle as well. It is recommended that the needles should not be reused [26, 38]. This practice was followed by fewer numbers of nurses in this study. Reuse of needle is associated with contamination and infection [38, 40] and increased risk of lipohypertrophy [41, 42]. Moreover, the risk of lipohypertrophy significantly increases with the use of needles for more than five times [41]. In the context of Nepal, it is a common practice to reuse needle by patients [39] but there is no evidence on the practice of safer ways to recap needles prior to disposal or for reuse from Nepalese setting. Systematic site rotation is crucial for preventing lipohypertrophy [41–43] and this recommendation was followed by most of the nurses in our study. Injecting insulin in lipohypertrophy areas reduces insulin absorption and action, leading to higher postprandial glucose level. Moreover, injecting insulin in lipohypertrohy areas causes significant intrasubject variability in its absorption and action [44]. In present study, about 30% of nurses also reported that they use single insulin pen for two different cartridges. This practice sometimes causes poor glycemic control if they do not consider the priming of insulin pen before injection and the compatibility of the pen for two different cartridges [45]. Results of this study highlight the need for urgent educational intervention for nurses on insulin injection technique through insulin pen. The finding of educational intervention has also been reported to be encouraging [46].

To our knowledge, this is the first study to report the insulin injection practice of nurses through insulin pen from Nepal. However, this study was limited to a single center, conducted on limited number of nurses, and thus the findings might not be generalizable. This study used self-administered questionnaire, therefore the results might defer from actual practice. Also, this study did not investigate nurses' rationale for their practices.

## 5. Conclusion

The insulin injection practice of nurses was suboptimal and highlights the need for urgent educational intervention among younger nurses and those with limited work experiences, though practice in senior nurses needs further exploration. Moreover, workplace-based learning and point-of-care interventions need to be considered for improving current practice.

## Data Availability

The datasets used and/or analyzed during the current study are available from the corresponding author on reasonable request.
